# Pharmacological Inhibition of Spermine Oxidase Reduces Neurodegeneration and Improves Retinal Function in Diabetic Mice

**DOI:** 10.3390/jcm9020340

**Published:** 2020-01-25

**Authors:** Fang Liu, Alan B. Saul, Prahalathan Pichavaram, Zhimin Xu, Madhuri Rudraraju, Payaningal R. Somanath, Sylvia B. Smith, Ruth B. Caldwell, S. Priya Narayanan

**Affiliations:** 1 Clinical and Experimental Therapeutics, College of Pharmacy, University of Georgia, Augusta, GA 30912, USA; fliu1@augusta.edu (F.L.); mrudraraju@augusta.edu (M.R.); sshenoy@augusta.edu (P.R.S.); 2 Culver Vision Discovery Institute, Augusta University, Augusta, GA 30912, USA; asaul@augusta.edu (A.B.S.); Prahalathan.Pichavaram@STJUDE.ORG (P.P.); zhxu@augusta.edu (Z.X.); sbsmith@augusta.edu (S.B.S.); rcaldwel@augusta.edu (R.B.C.); 3 Vascular Biology Center, Augusta University, Augusta, GA 30912, USA; 4 Charlie Norwood VA Medical Center, Augusta, GA 30904, USA; 5 Department of Ophthalmology, Medical College of Georgia, Augusta University, Augusta, GA 30912, USA; 6 Department of Cellular Biology and Anatomy, Medical College of Georgia, Augusta University, Augusta, GA 30912, USA

**Keywords:** diabetes, diabetic retinopathy, spermine oxidase, neurodegeneration, polyamine metabolism, MDL 72527, retinal ganglion cells

## Abstract

Diabetic retinopathy (DR) is a significant cause of blindness in working-age adults worldwide. Lack of effective strategies to prevent or reduce vision loss is a major problem. Since the degeneration of retinal neurons is an early event in the diabetic retina, studies to characterize the molecular mechanisms of diabetes-induced retinal neuronal damage and dysfunction are of high significance. We have demonstrated that spermine oxidase (SMOX), a mediator of polyamine oxidation is critically involved in causing neurovascular damage in the retina. The involvement of SMOX in diabetes-induced retinal neuronal damage is completely unknown. Utilizing the streptozotocin-induced mouse model of diabetes, the impact of the SMOX inhibitor, MDL 72527, on neuronal damage and dysfunction in the diabetic retina was investigated. Retinal function was assessed by electroretinography (ERG) and retinal architecture was evaluated using spectral domain-optical coherence tomography. Retinal cryosections were prepared for immunolabeling of inner retinal neurons and retinal lysates were used for Western blotting. We observed a marked decrease in retinal function in diabetic mice compared to the non-diabetic controls. Treatment with MDL 72527 significantly improved the ERG responses in diabetic retinas. Diabetes-induced retinal thinning was also inhibited by the MDL 72527 treatment. Our analysis further showed that diabetes-induced retinal ganglion cell damage and neurodegeneration were markedly attenuated by MDL 72527 treatment. These results strongly implicate SMOX in diabetes-induced retinal neurodegeneration and visual dysfunction.

## 1. Introduction

Diabetic retinopathy (DR), is a significant public health problem in the US and is the leading cause of blindness in working aged adults. The vision loss in DR patients results from diabetes-induced progressive retinal damage. The paucity of effective treatments for DR is a significant clinical problem. Even though DR is now recognized as a neurovascular disease [1,2,3,4], the existing therapies target vascular complications associated with late stages of the disease, moreover, they have unfavorable side effects. Several reports have shown that retinal neurons become dysfunctional and undergo degeneration during the initial stages of diabetes [5,6,7]. 

The characteristic features of diabetes-induced neurodegeneration in the retina include neuronal dysfunction, retinal thinning and apoptosis of retinal neurons. Impairment of the ERG response is a major feature of DR in patients and pre-clinical models [8,9]. Changes in ERG responses have been detected in diabetic patients even before the microvascular alterations are observed [10,11]. Several studies have reported the loss of RGCs in diabetic patients [12,13,14], and animal models [15,16]. It has been reported that the earliest cell types to undergo cell death in the diabetic retina are the RGCs [17,18]. Progressive thinning of the retinal layers is another major feature of DR [19,20]. Several studies have confirmed structural changes in experimental models of diabetes, diabetic patients, and human retinas from post-mortem samples [12,21,22]. Further studies on the mechanisms underlying diabetes-induced retinal neuronal damage and dysfunction are in great need of identifying new therapeutic targets for DR. 

The polyamine metabolic pathway is exquisitely regulated by the combined actions of multiple enzymes. Deregulation of the polyamine metabolism is shown to be associated with various neurodegenerative disease conditions such as Alzheimer’s disease, [23,24,25] Parkinson’s disease, [26,27,28] traumatic brain injury [29], and ischemic brain damage [30,31,32]. Alterations in polyamine metabolism cause cellular damage and cell death through the generation of oxidative byproducts [33]. Reports from our laboratory are the first to document that polyamine oxidase function is critically involved in causing neuronal dysfunction and vascular defects in the retina [34,35]. Spermine oxidase (SMOX) is a highly inducible enzyme in the polyamine signaling pathway. Dysregulation of SMOX can cause changes in cellular polyamine levels. Earlier studies from our laboratory have shown that SMOX expression is increased in models of hyperoxia-induced retinal degeneration and excitotoxicity-induced retinal neuronal damage [34,36]. Treatment with MDL 72527, a polyamine oxidase inhibitor, significantly improved neuronal survival and reduced retinal degeneration in both models. MDL 72527 is a widely used competitive inhibitor of SMOX and acetyl polyamine oxidase (commonly known as the polyamine oxidases) [37,38]. Neurodegeneration is an early event in DR. Even though the fundamental role of polyamine metabolism in neurodegenerative diseases has been considerably addressed, the impact of polyamine oxidation and its contribution to retinal neuronal damage induced by diabetes has not been studied. Utilizing MDL 72527, the present study investigated the impact of SMOX blockade in reducing diabetes-induced retinal neurodegeneration and dysfunction.

## 2. Materials and Methods

### 2.1. Animals 

All animal procedures were conducted in accordance with the ‘ARVO Statement for the Use of Animals in Ophthalmic and Vision Research’. All procedures were performed according to the approved institutional guidelines (Animal Welfare Assurance no. A3307–01) and adhered to the Public Health Service Policy on Humane Care and Use of Laboratory Animals (revised July 2017). We used C57BL6J male mice (Jackson Laboratories, Bar Harbor, ME, USA) in our experiments, and assured the minimum possible suffering during experimental procedures.

### 2.2. Induction of Diabetes

Eight-week-old male mice were chosen to induce diabetes according to our previously published method [39], by repeated intraperitoneal streptozotocin injections (up to 4 times) at a dose of 65 mg/kg (dissolved in 0.1 M sodium citrate buffer, pH 4.5). Control group received citrate buffer injections. Mice with blood glucose levels (determined using Alpha TRAK2 blood glucose monitoring system) higher than 350mg/dL were considered diabetic and maintained until 16 weeks post diabetic.

### 2.3. MDL 72527 Treatment

Animals (diabetic or controls) were treated with MDL 72527 (the SMOX inhibitor), administered intraperitoneally at a dose of 20 mg/kg in saline, three times a week until they were euthanized. Animals in the diabetic group received MDL 72527 treatment immediately after the onset of diabetes. Control groups received intraperitoneal injections of saline.

### 2.4. Immunofluorescence Staining

Retinal cryosections were immunostained according to our recently published methods [36,40]. The enucleated eyes were fixed overnight in 4% PFA at 4 °C, washed in PBS, cryoprotected in 30% sucrose and snap-frozen in optimal cutting temperature (OCT) solution. Cryosections (10 µm) were permeabilized in Triton X-100 (0.05%), blocked in normal goat serum (10% NGS, for 1 h), and incubated in respective primary antibodies (Table 1) at 4 °C overnight, followed by PBS wash and incubation (1 h) with the fluorescein-conjugated secondary antibody. The sections were rinsed in PBS and mounted (Vectashield, Vector Laboratories cat. no. H-1000, Burlingame, CA, USA). Image acquisition was performed using a confocal microscope (LSM 780; Carl Zeiss, Thornwood, NY, USA). Tuj1 intensity was quantified using Image J software. 

### 2.5. Quantification of RGCs and Fluorescence Intensity of Conjugated Acrolein

Confocal images (20X) were taken at 500 µm from the optic nerve and the number of Brn3a positive cells on the GCL or the fluorescence intensity of conjugated acrolein were quantified using Image J software. A minimum of three sections (20 µm apart) per retina were imaged and used for quantification studies.

### 2.6. Western Blotting

Immunoblotting experiments were performed as previously described [36]. Whole retinal tissues (from control and diabetic mice) were isolated and homogenized using RIPA buffer (Millipore, Billerica, MA, USA) consisting of protease (Complete Mini) and phosphatase (phosSTOP, Roche Applied Science, Indianapolis, IN, USA) inhibitor cocktails. Pierce^TM^ BCA protein assay (Thermo Scientific, Rockford, IL, USA) was used for the estimation of protein concertation. Around 20 ug protein per sample was used for Western blotting analysis. Samples were subjected to SDS-PAGE and transferred to nitrocellulose membrane (Millipore, Billerica, MA, USA). The membranes were blocked with non-fat dry milk (5%), incubated overnight in respective primary antibody (Table 1) at 4 °C, treated with anti-rabbit or anti-mouse HRP-conjugated secondary antibodies (GE-Healthcare, Piscataway, NJ, United States), followed by detection using enhanced chemiluminescence system (GE-Healthcare, Piscataway, NJ, USA). Densitometry analysis was using ImageJ software and normalized to loading controls.

### 2.7. Spectral Domain-Optical Coherence Tomography (SD-OCT)

Retinal structural integrity was assessed using SD-OCT by measuring the thickness of the retina and retinal layers. Ketamine/xylazine (73 mg/kg ketamine hydrochloride and 7.3 mg/kg xylazine hydrochloride, i.p.) were used to anesthetize the mice. Pupils were dilated with 1% tropicamide (Bausch & Lomb, Tampa, FL, USA), followed by the application of GenTeal Lubricant Eye Gel (Alcon, FortWorth, TX, USA). To keep the cornea moist throughout the procedure, Systane lubricant eye drops (Alcon) was applied. The Bioptigen Spectral Domain Ophthalmic Imaging System, SDOIS (Bioptigen Envisu R2200, Morrisville, NC, USA) was used as described previously [40,41]. The thickness of the retinal layers was generated using DIVERS software included with the instrument.

### 2.8. Electroretinogram (ERG) Analysis

Functional studies using ERG were conducted according the previously published method [42]. The animals were dark-adapted overnight, prior to the ERG experiment. Under dim red light, animals were anesthetized with ketamine and xylazine. Corneas were treated with proparacaine (0.5%), and pupils dilated with topical phenylephrine HCl (2.5%) and tropicamide (1%). A rectal probe connected to a heating pad was used to maintain the body temperature at 37 °C. A ground electrode was placed in the tail, and reference electrodes in each cheek. Silver thread electrodes were placed on each eye, and to improve electrical contact and protect the cornea from drying, a drop of hypromellose was added. Optic fibers were then positioned just in front of each pupil, leading the light from an LED device to the eyes. In order to provide extremely dim flashes, ranging from 2 × 10^−8^ to 10^−6^ scotopic lumens, the light from a blue (470 nm) LED was defocused and filtered before arriving at the optic fiber launcher. Testing consisted of a set of 5 ms flashes over a range of intensities, randomly interleaved with a probability distribution emphasizing intensities just above threshold (which is around 10^−8^ lumens). Over 10–100 trials, responses were averaged at each intensity, and positive (pSTR) and negative (nSTR, data not shown) scotopic threshold responses were measured at 110 and 200 ms, respectively, after the flash that occurred 500 ms into each 2 s trial. The STR amplitudes had floors at 0 μV. The results were averaged across the two eyes of each mouse, and across the mice in each group (treated and untreated control and diabetic), and the differences between the treated and untreated diabetic eyes were used to estimate the effects of the MDL 72527 treatment.

### 2.9. Statistical Analysis

Unless otherwise stated, One-way ANOVA, using GraphPad Prism software, was performed in all analyses. The post hoc test was Tukey’s test. *p* ≤ 0.05 was considered statistically significant.

## 3. Results

### 3.1. Effect of MDL 72527 Treatment on Body Weight and Blood Glucose

**Figure 1:**
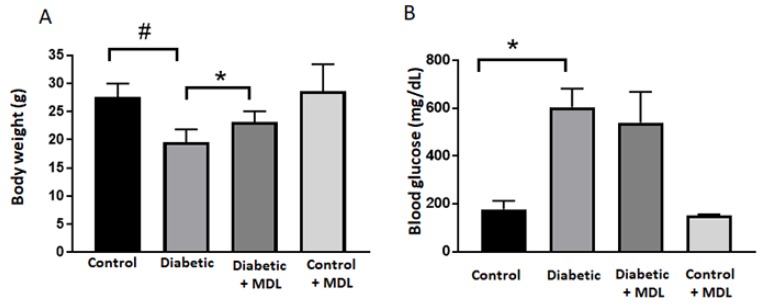
Changes in blood glucose and body weight. Bodyweight (**A**) and blood glucose levels (**B**) are recorded at the time of sacrifice, in groups of control, diabetic, diabetic+ MDL, and control+ MDL. Data represented as mean ± SD. * *p* < 0.05; # *p* < 0.01. *N* = 6–12 per group.

Bodyweight and blood glucose levels were measured in the diabetic mice (16 weeks post diabetic) and respective control groups at the time of sacrifice (Figure 1). The average weight of control mice was around 27.6 g, and the blood glucose averaged less than 200mg/dL. A significant reduction in body weight (*p* < 0.001) and a considerable increase in blood glucose levels (*p* < 0.05) were observed in the STZ diabetic mice. As shown in Figure 1A, MDL 72527 treatment significantly reduced the weight loss in diabetic mice but had no significant effect on the diabetes-induced increase in the blood glucose level (Figure 1B). 

### 3.2. The Expression of SMOX Is Increased in the Diabetic Retina

**Figure 2:**
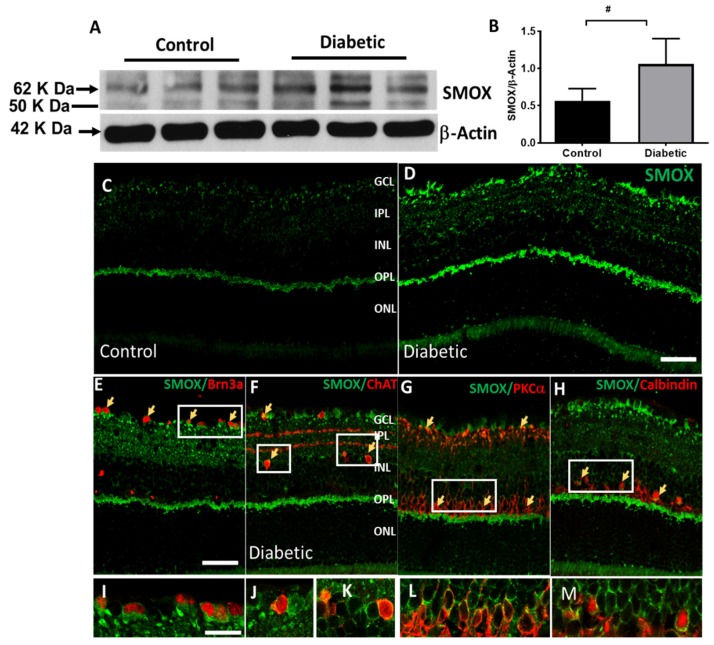
Increased spermine oxidase (SMOX) expression in the diabetic retina. (**A,B**) Western blot analysis and quantification showing elevated expression of SMOX in the 4 weeks diabetic retina. Data represented as mean ± SD. ^#^ *p* < 0.01. *N* = 6 per group. (**C,D**) Confocal images showing SMOX expression on control and diabetic retinal sections. Increased expression of SMOX in the ganglion cell layer (GCL), inner nuclear layer (INL), and outer plexiform layer (OPL) of the diabetic retina. (**E–H**) Colocalization studies showing SMOX expression in retinal ganglion cells (Brn3a positive), amacrine cells (ChAT positive), bipolar cells (PKCα positive), and horizontal cells (calbindin positive). Areas of colocalization are represented by arrows. Scale bar 50 µm. (**I–M**) High magnification images of SMOX colocalization in the diabetic retina. Scale bar 20 µm. *N* = 4–6 were included per group, and representative images are presented.

Western blotting and immunofluorescence methods were utilized to determine the expression of SMOX in diabetic retina. A significant upregulation in SMOX protein level was observed in the STZ-diabetic mouse retinas (4 weeks post diabetic) as compared to controls (Figure 2A,B). Immunolabeling analysis showed increased expression of SMOX in the ganglion cell layer (GCL), outer plexiform layer (OPL), and to some extent, in the inner nuclear layer (INL) (Figure 2C,D). Colocalization studies (2E–H) showed that SMOX is expressed in Brn3a-positive ganglion cells, PKCα− positive bipolar cells, ChAT- positive amacrine cells, and calbindin-positive horizontal cells. These results are consistent with our previous studies demonstrating an increase in SMOX expression in ischemic retinopathy models [43].

### 3.3. Inhibition of SMOX with MDL 72527 Preserved Inner Retinal Function in the Diabetic Mice

**Figure 3:**
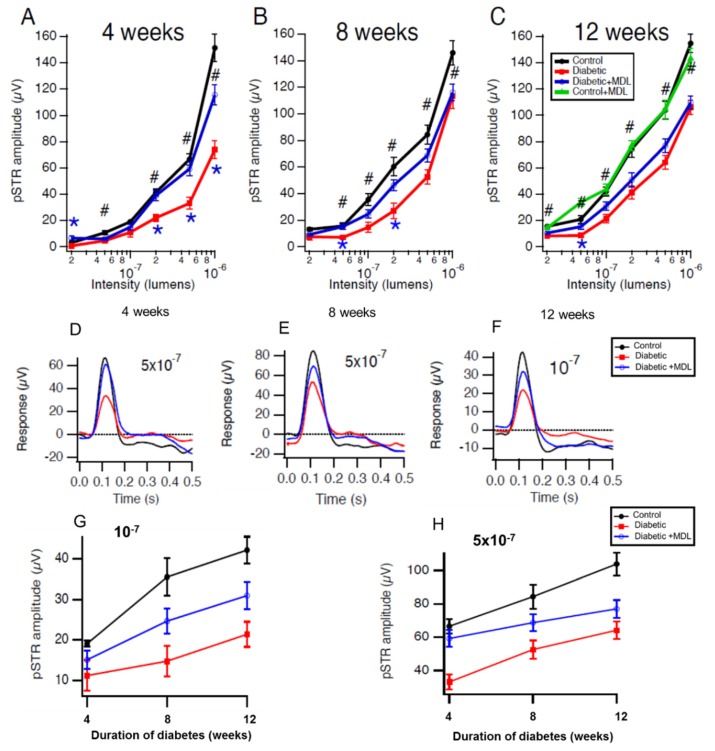
Treatment with MDL 72527 preserved inner retinal function in the diabetic mice. (**A**–**C**) Studies by dark-adapted electroretinography showing the positive scotopic threshold responses (pSTRs, a sensitive measure of inner retinal function). The pSTRs are significantly reduced in diabetic animals (4, 8, and 12 weeks post diabetic). Treatment with MDL 72527 preserved the pSTR amplitudes in the diabetic group showing a partial rescue of inner retinal function with SMOX inhibition. Responses from vehicle-treated and MDL 72527 treated controls show no differences in their pSTRs. Significant differences at individual intensities are shown (t-tests with Holm-Bonferroni corrections for multiple comparisons; # *p* < 0.001, * *p* < 0.05). (**D**–**F**) Representative data showing responses at a single intensity in mice at 4, 8, and 12 weeks post diabetes. (**G**,**H**) Summarizing the effects of duration of diabetes on pSTRs (at two different intensities). * diabetic vs diabetic +MDL, # diabetic vs Control. Number of animals per group are: Control (4), Diabetic (4), and Diabetic +MDL (7) for 4 weeks; Control (7), Diabetic (8), and Diabetic +MDL (12) for 8 weeks and Control (11), Diabetic (11), Diabetic +MDL (12) and Control +MDL (4) for 12 weeks study.

Alterations in retinal function have been demonstrated in DR patients and animal models of DR. Utilizing ERG analysis, we investigated the impact of SMOX inhibition on diabetes-induced functional changes in the retina. Positive scotopic threshold responses (pSTRs) studied by dark-adapted electroretinography showed significant reductions in STZ-diabetic mice at 4, 8 and 12 weeks after the onset of diabetes, compared to the non-diabetic control group (Figure 3A–C). Our results show that treatment with MDL 72527 markedly improved diabetes-induced functional defects in diabetic mice at all the stages studied. MDL 72527-mediated preservation of retinal function is more evident in the earlier stages of diabetes studied. Compared to STZ treated diabetic mice, SMOX inhibition by MDL significantly improved pSTRs at four different light intensities in 4 weeks post diabetic mice (Figure 3A) and at two different intensities in 8 week post diabetic mice (Figure 3B). The responses in 12 week post diabetic mice showed an improvement in response to MDL 72527 treatment. This, however, was statistically significant at only one intensity (Figure 3C). Responses from MDL 72527 treated non-diabetic control groups (12 weeks) did not show any difference compared to vehicle-treated controls. Representative responses at single intensities at three different duration of diabetes are shown in Figure 3D–H. A summary of the effects of the duration of diabetes on pSTRs at two different intensities (10^−7^ and 5 × 10^−7^ lumens) is presented in Figure 3G and H. It is evident in all the 3 groups that pSTR responses increased with age; however, induction of diabetes reduced the responses at all the intensities studied. Treatment with MDL 72527 improved the pSTR responses in STZ diabetic mice, at all the intensities studied (Figure 3G–H). 

### 3.4. In Vivo Evaluation of Retinal Architecture

**Figure 4:**
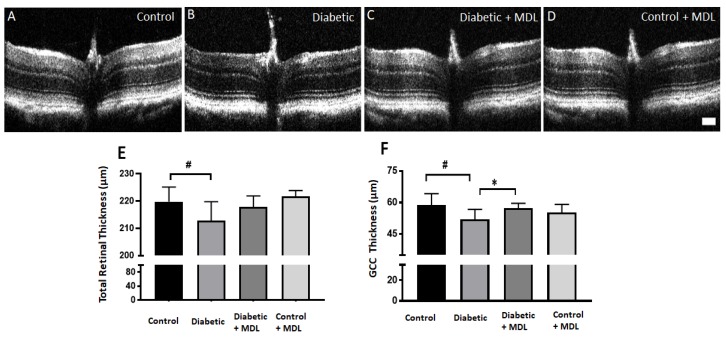
Analysis of diabetes-induced retinal thinning. (**A**–**D**) Representative images (B-scan) obtained from the spectral domain optical coherence tomography (SD-OCT) analysis of control, diabetic, MDL 72527-diabetic and MDL 72527-treated control retinas. (**E**–**F**) Quantification of the retinal thickness (from NFL to outer segment/RPE interface) and thickness of the ganglion cell complex (RNFL+GCL+IPL) showing a significant decrease in diabetic retinas compared to controls. MDL 72527 treatment significantly improved diabetes-induced thinning of the inner retina. Results presented as mean ± SD. *N* = 5–12 per group. # *p* < 0.01, * *p* < 0.05. scale bar 100 µm.

**Figure 5:**
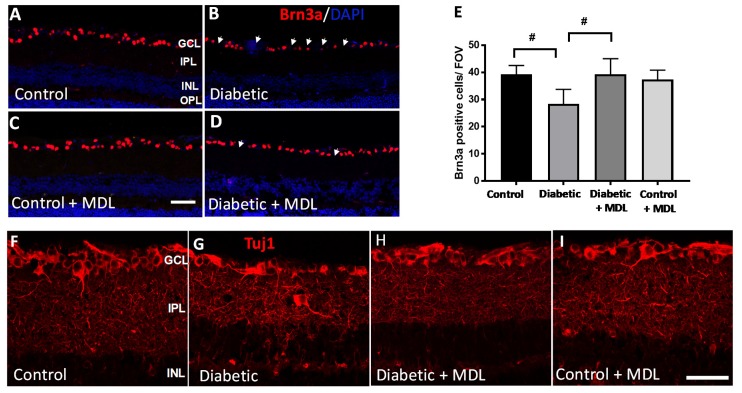
Effect of MDL 72527 treatment on diabetes-induced RGC loss. (**A**–**D**) Immunostaining of retinal cryostat sections using Brn3a antibody showing a reduced number of Brn3a positive RGCs in the diabetic retina (16 weeks), compared to control. Treatment with MDL 72527 improved the survival of Brn3a positive cells. (**E**) Quantitative analysis demonstrating significant loss of Brn3a-positive cells in the GCL in response to diabetes. Treatment with MDL 72527 protected against the diabetes-induced RGC loss. (**F–I**) Immunostaining using Tuj1 marker showing the axonal loss in diabetic retina compared to controls. MDL 72527 treatment reduced axonal degeneration in the diabetic retina. Treatment with MDL 72527 markedly improved Tuj1 levels in diabetic retina. *N* = 5–6 per group were included in the experiment and representative images are shown. Data are presented as mean ± SD. Data are presented as mean ± SD. # *p* < 0.01. Scale bar 50 µm.

Diabetes induced retinal thinning is reported in experimental models and patients [21]. In the current study, the thickness of retinal layers was quantified in groups of mice (15 weeks post diabetic) and their respective controls, using SD-OCT (Figure 4A–D). Similar to previous reports [44] diabetic mice exhibited significant retinal thinning as compared to the control group. Diabetes induced retinal thinning was evident in STZ- diabetic mice compared to non-diabetic controls. The thinning was pronounced in the thickness of the total retina and the ganglion cell complex (GCC, RNFL+GCL+IPL). Mice treated with MDL 72527 showed preservation of total retinal thickness compared to diabetic controls (Figure 4E), however this change was not statistically significant. However, the improvement observed in the GCC thickness was significant in the MDL 72527 treated group (Figure 5F). No significant differences were observed in the measurements of OPL, ONL, or RPE thickness across the groups studied.

### 3.5. Treatment with MDL 72527 Reduced the Loss of RGCs in the Diabetic Retina

RGC degeneration is a significant feature of DR. In the current study, diabetes-induced RGC loss was investigated using immunofluorescence staining of Brn3a, an RGC marker using retinal cryosections. Representative images of retinal sections immunostained using Brn3a (Figure 5A–D) showed a significant reduction in Brn3a positive neurons in the diabetic retinas. Treatment with MDL 72527 improved the survival of RGCs in the diabetic retina. Quantification of Brn3a was performed on retinal cryostat sections using Image J software (Figure 5E) in GCL. The analysis demonstrates a significant loss of Brn3a-positive cells in the GCL in response to diabetes. Treatment with MDL 72527 significantly protected against diabetes-induced RGC loss.

Immunofluorescence analysis of retinal sections (16 weeks post diabetic) using Tuj1 (Figure 5F–I) provided additional evidence towards diabetes-induced degeneration of retinal neurons. A marked reduction in the Tuj1 (beta III Tubulin that stains RGC axons) immunostaining in STZ -diabetic retinas indicated the axonal loss due to the induction of diabetes. Treatment with MDL 72527 improved Tuj1 expression in the diabetic retinas.

### 3.6. Diabetes-Induced Neurodegeneration is Reduced with MDL 72527 Treatment

**Figure 6:**
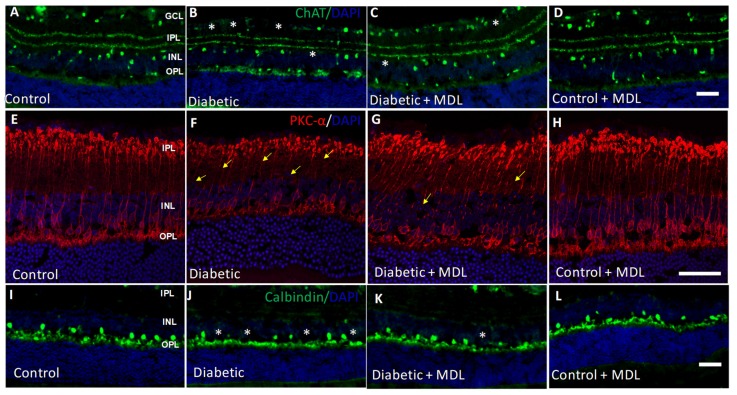
Treatment with MDL 72527 reduced neurodegeneration in the diabetic retina. (**A**–**D**) Immunostaining of retinal cryostat sections using the ChAT antibody showing loss of amacrine cells in the diabetic retina (16 weeks), compared to control. (**E**–**H**) Immunostaining using PKCα, a marker for rod bipolar cells demonstrates the presence of degenerating axons and synaptic ends in the diabetic retina. (**I**–**L**) Immunofluorescence images showing the loss of horizontal cells in the diabetic retina by calbindin immunoreactivity. MDL 72527 treatment markedly reduced the neurodegenerative changes. Asterisks indicate areas of cell loss, and arrows indicate areas of axonal degeneration. *N* = 5–6 per group and representative images are presented. Scale bar 50 µm.

In addition to RGCs, other retinal neurons also undergo degeneration in response to diabetes. In the present study, we investigated changes in retinal bipolar, amacrine and horizontal cells. Our qualitative analysis as presented in Figure 6, demonstrates that diabetes-induced degenerative changes are evident in STZ retinas compared to the controls. Immunostaining using the ChAT antibody showed a decrease in the number of amacrine cells in the diabetic retina compared to controls, while MDL treatment reduced this change (Figure 6A–D). Immunofluorescence studies (Figure 6E–H) using PKCα, a marker for rod bipolar cells in control and diabetic retinas treated with or without MDL. Degenerative changes such as shorter and distorted processes, and fewer cell bodies are evident in the diabetic retinas (Figure 6F), treatment with MDL 72527 improved these alterations (Figure 6G). The presence of horizontal cells was decreased in the diabetic retina, as studied by calbindin immunostaining, while MDL treatment improved the survival of these in the diabetic retina (Figure 6I–L). Treatment with MDL 72527 reduced these neurodegenerative changes in the diabetic retina. 

### 3.7. Treatment with MDL 72527 Reduced Conjugated Acrolein Levels in the Diabetic Retina

**Figure 7:**
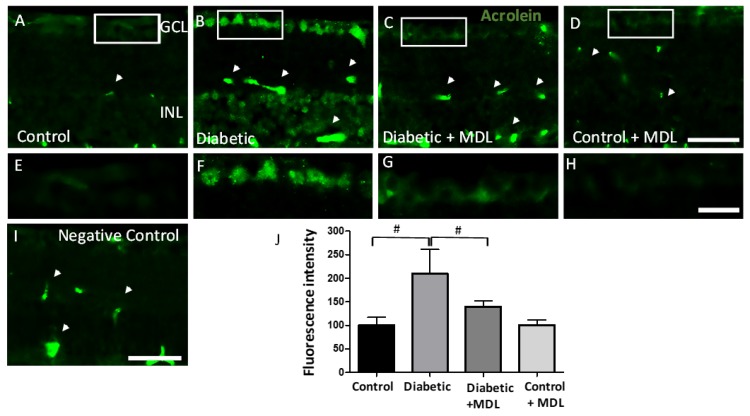
Effect of MDL 72527 treatment on the conjugated acrolein levels in the diabetic retina. (**A**–**D**) Immunofluorescence studies on retinal cryostat sections using conjugated acrolein antibody showing increased levels in the GCL and INL of diabetic retina, compared to control. Treatment with MDL 72527 reduced the conjugated acrolein levels in the diabetic retina. Scale bar 50 um. (**E**–**H**) High magnification images showing acrolein in the GCL of retinal sections. Scale bar 20 µm. (**I**) Negative control using secondary antibody. Arrow heads represent areas of non-specific labelling (**J**) Quantitative analysis demonstrating significant upregulation of acrolein in response to diabetes, while treatment with MDL 72527 reduced the changes. *N* = 4–6 per group and results presented as mean ± SD. # *p* < 0.01.

Acrolein, a highly reactive aldehyde and a potent mediator of oxidative damage is a major downstream effector of SMOX function. In the present study, using immunofluorescence experiments, we investigated the impact of SMOX inhibitor, MDL 72527 on the levels of conjugated acrolein in the diabetic retina (Figure 7). An elevated level of conjugated acrolein is evident in the GCL and INL of the diabetic retina (Figure 7A,B), while MDL 72527 treatment reduced the diabetes-induced upregulation of conjugated acrolein (Figure 7C,D). High magnification images of conjugated acrolein in the GCL are shown in Figure 7E–H. Quantification of the fluorescence intensity on retinal sections showed a significant upregulation in the diabetic retina compared to non-diabetic controls, which is significantly reduced in response to MDL 72527 treatment (Figure 7G). The nonspecific staining observed in the retinal sections (as seen in the negative controls, Figure 7I) were subtracted during quantification. These results support the involvement of acrolein induced cellular damage as a potential mechanism of SMOX regulated neurodegeneration in the diabetic retina.

## 4. Discussion

Neurodegeneration is an early event in the diabetic retina. Diabetes-induced neuronal damage is characterized by reduced retinal neuronal function, neuronal cell death, and thinning of the inner retina and nerve fiber layers [45,46,47]. Nevertheless, the molecular mechanisms mediating these events are not clearly understood. We recently showed that oxidation of polyamines is elevated during hyperoxia-induced retinal degeneration [34] and that MDL 72527 treatment significantly decreased the retinal neuronal death in the models of oxygen-induced retinopathy and excitotoxicity [34,35,36]. Here, we present the neuroprotective effect of MDL 72527 on diabetes-induced retinal neuronal damage and dysfunction. Our current study is the first report examining the impact of SMOX inhibition in preventing diabetes-induced neurodegeneration in the retina.

Blockade of polyamine oxidases using MDL 72527 has shown to significantly reduce brain edema and ischemic injury in a rat model of cerebral ischemia [38], and is neuroprotective after traumatic brain injury in an experimental model [48]. So far, no studies have been reported on the impact of MDL 72527 treatment in animal models of diabetes. Altered levels of serum SMOX in patients with insulin-dependent diabetes mellitus and microvascular complications have been reported previously [49]. Altered levels of polyamine have also been reported in the vitreous samples from PDR patients [50]. Our present study demonstrating increased SMOX expression in the diabetic retina is consistent with the earlier studies from our laboratory showing elevated SMOX levels in the OIR retina [34] and the NMDA-induced excitotoxicity model [36]. SMOX is reported as a crucial enzyme in the polyamine catabolic pathway which plays a significant role in maintaining the polyamine homeostasis [27,51]. The involvement of SMOX in neurodegenerative diseases has been reported by other laboratories [52,53]. Elevated SMOX/APAO levels represent elevated oxidative stress resulting from elevated polyamine oxidation. [54]. Despite the efforts implicating polyamine oxidation in neurodegenerative diseases, such an event in DR has not yet been demonstrated. 

Alterations in ERG responses are a major characteristic feature of DR. Previous studies have shown that retinal function is severely affected in DR patients and experimental models [8,9]. Our results demonstrate functional deficits in the diabetic retina as early as four weeks of diabetes. ERG alterations have been observed in streptozotocin (STZ) treated diabetic rats as early as two weeks after the onset of diabetes [55], four weeks in STZ-diabetic mice [56], and three months in the spontaneously diabetic Ins2Akita model [57]. Reduced b-wave amplitude has been reported in STZ diabetic mice, four months after the induction of diabetes [58] and in STZ-induced diabetic rats after seven weeks of diabetes [59]. The neuroprotective effect of MDL 72527 treatment in diabetes-induced retinal dysfunction is confirmed by the ERG analysis showing improved retinal function in diabetic mice. Our studies demonstrate that diabetes-induced reductions in pSTRs are also improved in response to MDL 72527 treatment. The origin of the pSTR in mice is thought to be predominantly retinal ganglion cells [60]. However, at the highest intensities used here, the response is dominated by the b-wave that arises from rod bipolar cells [61].

Progressive retinal thinning is another characteristic feature of DR. Several studies have reported diabetes-induced structural changes in retina, of patients, in the post-mortem human retinas and in experimental models of DR [12,21,22]. Our results from the SD-OCT analysis demonstrating reductions in total and inner retinal thickness are in support of diabetes-induced retinal thinning. A significant reduction in the thickness of the inner retina was observed between 3-6 weeks of diabetes in the Ins2Akita model [57] and in STZ- induced diabetic mice during five weeks post-onset of diabetes[62]. Thinning of the inner retina or NFL has also been reported in diabetic patients prior to the onset of DR and even before any visible vascular signs of DR appear, supporting the early onset of neurodegeneration in the diabetic retina and warranting neuroprotective intervention to prevent chronic neurodegeneration [63,64].

Several studies have documented the loss of RGCs in diabetic patients [12,13,14], and animal models [15,16]. It has been demonstrated that RGCs are the earliest cells to undergo cell death in the diabetic retina [17,18]. Furthermore, other degenerative changes such as loss of synapses, axonal beading, and morphological changes are also evident in the diabetic retina. In the present study, diabetes-induced RGC loss was evident in 16 weeks post diabetic retinas, while MDL 72527 treatment significantly reduced the RGC loss in the diabetic retina. We further noticed a reduction in Tuj1 expression, supporting axonal loss in the diabetic retina. Our qualitative studies also showed a decrease in amacrine and horizontal cells and evidence for degenerating bipolar cells in the diabetic retina. These changes were reversed by MDL 72527 treatment. Loss of amacrine and ganglion cells and severe deficits in synaptic connectivity are reported in the spontaneously diabetic Ins2Akita mice, 9 months of age [57]. A previous study conducted on the same model demonstrated dendritic abnormalities and RGC loss within 3 months of diabetes [7]. Similar findings are reported in STZ-induced diabetic (3 months) rats [65], followed by alterations in the photoreceptor layer and the retinal pigment epithelium (RPE) [66]. The neuroprotective effect of SMOX blockade in the diabetic retina is inconsistent with our previous report of the impact of MDL 72527 in limiting excitotoxicity-induced neuronal damage [36], one of the major mechanisms of neurodegeneration in the diabetic retina. Alterations in polyamines or their metabolites are also reported in vision disorders such as glaucoma [67], optic nerve injury [68], and experimental autoimmune encephalomyelitis (EAE) induced optic neuritis [69]. 

DR progression is further characterized by pathological features including inflammation to the retina, hypoxia, glial activation, vascular damages including increased vascular permeability and pathological angiogenesis [70,71,72]. Earlier studies from our group and others have reported the release of inflammatory cytokines and leukocyte adhesion to the capillaries in the diabetic retina [39], [73,74]. Diabetes-induced retinal vascular damages have been extensively studied by several laboratories [75,76,77]. Activation of the Muller glia is shown to be closely related to neurovascular changes in the diabetic retina [59,78,79,80]. However, since the focus of our present study is solely on diabetes-induced neurodegeneration in the retina, we have not evaluated these changes in response to SMOX inhibition.

**Figure 8:**
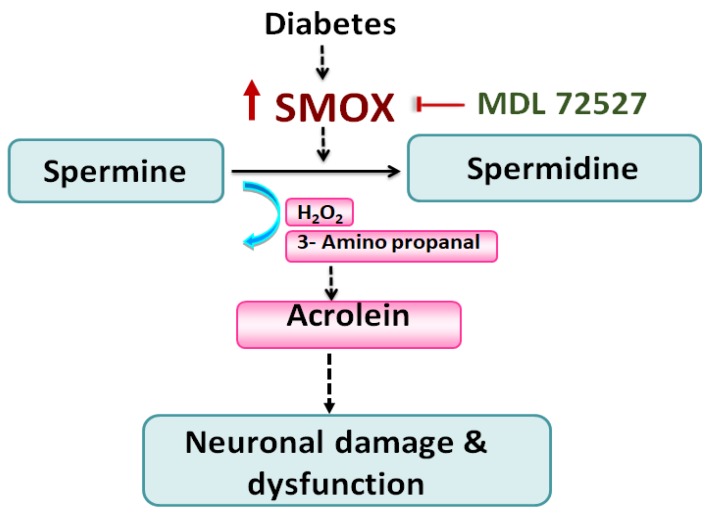
A proposed mechanism of SMOX-induced neurodegeneration in the diabetic retina. It is postulated that diabetes-induced upregulation of SMOX causes oxidation of spermine to spermidine, resulting in elevated levels of reactive aldehydes and H_2_O_2_. The reactive aldehyde, 3-aminopropanal (3-AP), gets converted to acrolein, a potent mediator of oxidative damage leading to neuronal damage and dysfunction in the retina. SMOX: Spermine oxidase; MDL 72527: N1, N4-bis(2,3-butadienyl)-1,4-butanediamine; H_2_O_2_: hydrogen peroxide.

SMOX is critically involved in the polyamine catabolism that plays an essential role in maintaining polyamine homeostasis [27,51]. Although the mechanisms by which SMOX contributes to neurodegeneration in DR is still not clear [45], Figure 8 depicts a probable hypothesis that warrants future investigation. Briefly, SMOX specifically regulates the oxidation of spermine to generate spermidine and at the same time produces hydrogen peroxide and 3-aminopropanal (3-AP) as byproducts possessing the potential of inducing cellular damage and pathologies [54,81,82]. Acrolein, a highly reactive aldehyde is generated by the spontaneous conversion of 3-AP formed by SMOX activity. Acrolein is highly toxic and a known potent mediator of oxidative modifications. Acrolein causes cellular damages by inducing inflammation, membrane disruption, protein adduction, endoplasmic reticulum stress, DNA damage, etc. [83]. Acrolein forms acrolein-lysine adducts, [N Nε- (3- formyl-3, 4-dehydropiperidino)] lysine called the FDP-Lysine [84]. Other studies have demonstrated the role of acrolein (generated during SMOX activity) in the progression of DR [59,85,86,87]. Using fibrovascular tissue from DR patients, a recent study documented FDP-Lys immunoreactivity in the CD34-positive cells and alpha-smooth muscle actin (α-SMA)-positive cells of the vascular compartment [86]. The serum and hemoglobin levels of FDP-lysine were significantly elevated in diabetic patients in comparison with control individuals. However, no significant association was observed between serum FDP-lysine levels and the severity of DR [88].Another study, using an experimental rat model of diabetes, demonstrated increased immunoreactivity of FDP-lysine in the Müller glia, where it initially accumulated within Müller glial end feet and thereafter spread distally throughout the inner radial processes of the cell [89]. In the present study, we investigated the changes in conjugated acrolein levels in the diabetic retina in response to MDL 72527 treatment. Our future experiments will include the evaluation of the changes in FDP-Lysine levels as well as acrolein-induced oxidative modifications in the diabetic retina.

## 5. Conclusions

The present study reports for the first time, the impact of blocking spermine oxidase, using systemic treatment with MDL 72527, in limiting diabetes-induced neurodegeneration in the retina. Our results demonstrate a crucial role for the SMOX pathway as one of the major mechanisms associated with diabetes-induced neuronal damage and dysfunction in the retina. Considering the need for new therapies for patients suffering from DR, our findings are clinically relevant. Our results suggest that blockade of spermine oxidase signaling can be considered as a therapeutic target to limit neuronal damage and dysfunction in vision disorders.

## Figures and Tables

**Table 1:** Antibodies used in the study.

Antibody	Cat. no.	Company	Dilution	Experiment
SMOX	15052-1-AP	Proteintech Group, Rosemont, IL, USA	1:200	Immunostaining
Brn3a	SC-31984	Santa Cruz, Dallas, TX, USA	1:200	Immunostaining
GFAP	Z0334	Dako, Carpinteria, CA, USA	1:200	Immunostaining
Tuj1	801202	BioLegend, San Diego, CA,USA	1:200	Immunostaining
Calbindin	C9848	Sigma-Aldrich, St. Louis, MO, USA	1:200	Immunostaining
ChAT	AB144P	Millipore, Billerica, MA, USA	1:200	Immunostaining
PKCα	11723	Abcam, Cambridge, UK	1:200	Immunostaining
SMOX	15052-1-AP	Proteintech Group, Rosemont, IL, USA	1:500	Western blotting
β-Actin	4511	Sigma-Aldrich, St. Louis, MO, USA	1:5000	Western blotting
Conjugated acrolein	ab48501	Abcam, Cambridge, UK	1:200	Immunostaining

## Abbreviations

APAO	Acetyl polyamine oxidase
ChAT	Choline acetyltransferase
DR	Diabetic retinopathy
EAE	Experimental autoimmune encephalomyelitis
ERG	Electroretinography
FDP lysine	3-formyl-3,4-dehydropiperidino lysine
GCL	Ganglion cell layer
HRP	Horse radish peroxidase
INL	Inner nuclear layer
NMDA	N-methyl D-aspartate
NGS	Normal goat serum
OCT	Optimal cutting temperature
OIR	Oxygen induced retinopathy
ONL	Outer nuclear layer
OPL	Outer plexiform layer
PBS	Phosphate buffer saline
PKC	Protein kinase Ca
RGC	Retinal ganglion cell
RPE	Retinal pigment epithelium
SD-OCT	Spectral domain-optical coherence tomography
SDS-PAGE	Sodium dodecyl sulphate-polyacrylamide gel electrophoresis
SMOX	Spermine oxidase
STR	Scotopic threshold response
STZ	Streptozotocin
Tuj1	Neuron-specific class III-beta tubulin
WT	Wild type
3-AP	3-Aminopropanal
